# High-Cost Medical Expense Benefit System in Japanese Universal Health Coverage

**DOI:** 10.31662/jmaj.2026-0085

**Published:** 2026-07-24

**Authors:** Takaaki Konishi, Kimiko Hirata, Mio Nakata, Mio Adachi, Keita Sasaki, Sumito Saeki, Naomi Sakurai, Yuko Tanabe, Eriko Tokunaga, Tsuguo Iwatani

**Affiliations:** 1 Division of Health Services Research, Institute for Cancer Control, National Cancer Center, Tokyo, Japan; 2 Department of Breast and Endocrine Surgery, Graduate School of Medicine, The University of Tokyo, Tokyo, Japan; 3 Department of Radiation Oncology, Osaka Red Cross Hospital, Osaka, Japan; 4 Department of Radiation Oncology, Osaka Medical and Pharmaceutical University, Takatsuki, Japan; 5 Department of Breast Surgery, Moriyama Memorial Hospital, Tokyo, Japan; 6 Japan Clinical Oncology Group Data Center/Operation Office, National Cancer Center Hospital, Tokyo, Japan; 7 Breast Surgical Oncology, Breast Oncology Center, Cancer Institute Hospital, Japanese Foundation for Cancer Research, Tokyo, Japan; 8 Cancer Survivors Recruiting Project, General Incorporated Association, Tokyo, Japan; 9 Department of Medical Oncology, Toranomon Hospital, Tokyo, Japan; 10 Department of Breast Oncology, NHO Kyushu Cancer Center, Fukuoka, Japan; 11 Department of Breast and Endocrine Surgery, St. Marianna University School of Medicine, Kawasaki, Japan

**Keywords:** catastrophic illness, cost sharing, health expenditures, insurance, universal coverage

## Abstract

Although the citizens of Japan have universal health coverage, the out-of-pocket expenses can still be exorbitant. In 1973, the High-Cost Medical Expense Benefit System was implemented to reduce this financial strain. The system was initially reimbursement based and later evolved into an in-kind benefit model, reducing upfront payments through prior applications or Individual Number Card use. The system permits expenses to be combined among household members; when several high costs have accrued, future monthly ceilings are reduced. Special provisions apply to chronic conditions such as dialysis and hemophilia. In 2024, a governmental proposal to raise the monthly ceiling of the High-Cost Medical Expense Benefit System, which was established to prevent individuals from incurring catastrophic health expenditures, faced criticism for excluding patient feedback. The government subsequently withdrew the proposal and formed a committee to discuss future reforms focusing on sustainability, intergenerational equity, and effective benefit delivery. The High-Cost Medical Expense Benefit System remains essential for ensuring access to advanced care without compromising patients’ daily lives.

## Introduction

The High-Cost Medical Expense Benefit System is the cornerstone of the public health insurance framework of Japan. This system mitigates the financial burden of medical care by capping monthly out-of-pocket expenditures. When a co-payment for insured medical services exceeds a predefined threshold (calculated based on age and income), the excess amount becomes eligible for reimbursement ^(1)^. The system operates within the universal health coverage model; that is, all residents must be enrolled in public health insurance, either through employment-based programs or National Health Insurance ^(2)^.

The High-Cost Medical Expense Benefit System prevents individuals from incurring catastrophic health expenditures ^(3)^. The design also reflects a deliberate effort to balance governmental financial protection with fiscal sustainability, especially in the context of the rapidly aging population and increasing healthcare costs ^(4)^. The Japanese government recently proposed raising the cap on monthly out-of-pocket payments to strengthen the financial sustainability of the health insurance system. However, the plan was ultimately put on hold due to strong opposition from patient groups ^(5)^, highlighting the political challenge of cost-sharing policies in a universal coverage system.

This article aims to explain the High-Cost Medical Expense Benefit System of Japan because it remains underexplored in English-language academic literature. Furthermore, we discuss policy-relevant design features of the system that may offer transferable insights for other universal health coverage settings.

## High-Cost Medical Expense Benefit System

The High-Cost Medical Expense Benefit System reimburses a portion of medical expenses that exceed a predefined monthly ceiling (Figure 1). The ceiling is determined based on the age and income of the patient (Table 1) ^(1)^. Out-of-pocket payments made by multiple family members under the same insurance system are combined each month (household aggregation). If the total amount exceeds the monthly ceiling, the excess amount is reimbursed. For individuals aged <70 years, only expenses exceeding ¥21,000 per medical institution can be combined. If a household reaches the monthly ceiling three or more times within 12 months, the ceiling amount is reduced from the fourth occurrence onward (multimonthly ceiling). Meal expenses during hospitalization and additional charges for a private room are not eligible for coverage. For example, a working-age household with an annual income of approximately ¥5 million undergoing repeated chemotherapy costing ¥300,000 per month would initially face a monthly out-of-pocket ceiling of ¥80,430 ([¥300,000 − ¥267,000] × 1% + ¥80,100); after reaching the ceiling three times, the multimonthly ceiling mechanism would apply, reducing subsequent out-of-pocket payments to ¥44,400.

**Figure 1.**
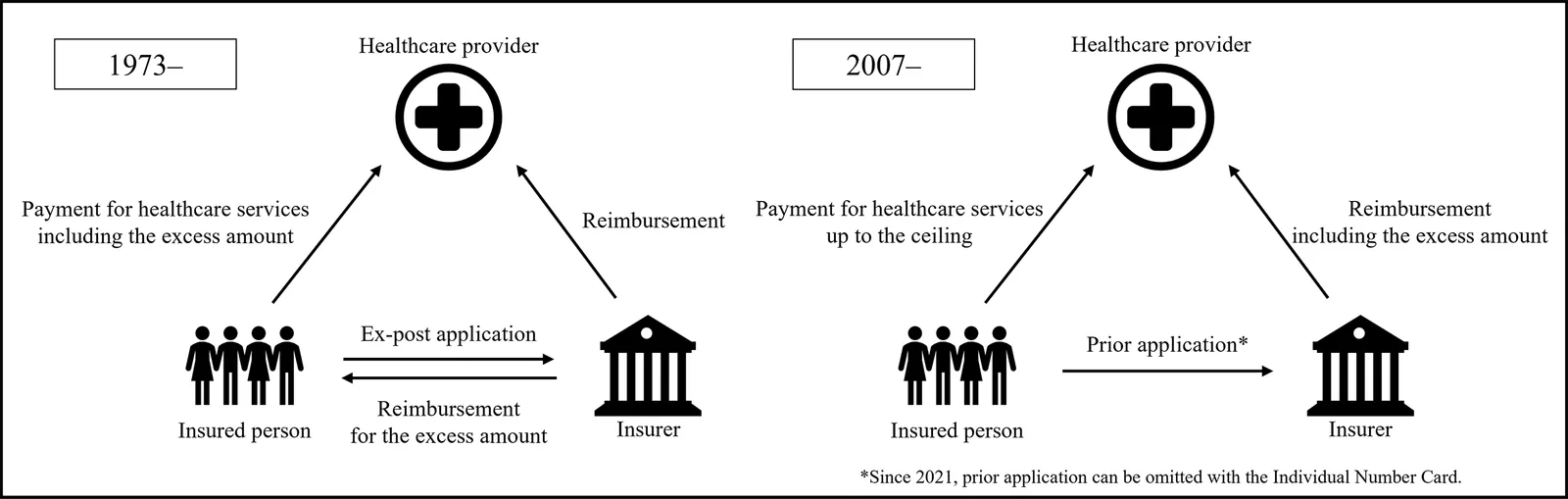
Flow of the High-Cost Medical Expense Benefit System. If the total payment exceeds the monthly ceiling defined by age and income as shown in Table 1, the excess is reimbursed. Since 2007, upon prior application to the insurer, patients have only been required to pay either their standard co-payment or the monthly ceiling amount, whichever is less (i.e., an in-kind benefit system). In 2021, an online qualification verification system was developed that enabled patients to scan their Individual Number Cards to receive in-kind benefits without prior application.

**Table 1.** Predefined Monthly Ceiling of the High-Cost Medical Expense Benefit System, 2018–Present.

Approximate annual income	Monthly ceiling per family	Multimonthly ceiling
Individuals aged <70 years		
≥¥11.6 million (≥$83,000)	(ME − ¥842,000) × 1% + ¥252,600 ([ME − $6,000] × 1% + $1,800)	¥140,100 ($1,000)
¥7.7–11.6 million ($55,000–83,000)	(ME − ¥558,000) × 1% + ¥167,400 ([ME − $4,000] × 1% + $1,200)	¥93,000 ($660)
¥3.7–7.7 million ($26,000–55,000)	(ME − ¥267,000) × 1% + ¥80,100 ([ME − $1,900] × 1% + $570)	¥44,400 ($310)
¥1.0–3.7 million ($7,000–26,000)	¥57,600 ($410)	¥44,400 ($310)
<¥1.0 million* (<$7,000*)	¥35,400 ($250)	¥24,600 ($180)
Individuals aged ≥70 years		
≥¥11.6 million (≥$83,000)	(ME − ¥842,000) × 1% + ¥252,600 ([ME − $6,000] × 1% + $1,800)	¥140,100 ($1,000)
¥7.7–11.6 million ($55,000–83,000)	(ME − ¥558,000) × 1% + ¥167,400 ([ME − $4,000] × 1% + $1,200)	¥93,000 ($660)
¥3.7–7.7 million ($26,000–55,000)	(ME − ¥267,000) × 1% + ¥80,100 ([ME − $1,900] × 1% + $570)	¥44,400 ($310)
¥1.6–3.7 million ($11,000–26,000)	¥57,600^†^ ($410^†^)	¥44,400 ($310)
<¥1.6 million* (<$11,000*)	¥24,600 or 15,000^‡^ ($180 or $110^‡^)	—^§^

Note: Currency conversions were based on an exchange rate of ¥140 = US $1. *Individuals with this annual income level are exempt from paying residential taxes. ^†^Regarding outpatient ME, the monthly ceiling was ¥18,000 ($130) per individual. ^‡^Regarding outpatient ME, the monthly ceiling was ¥8,000 ($57) per individual. ^§^Individuals in this category were ineligible for a multimonthly ceiling. ME: medical expenses.

To receive reimbursement, individuals must submit a claim to their health insurance provider along with the required documentation (e.g., receipts). Because time is required to verify the expenses, it can take approximately 3 months for claims to be processed. Claims can be submitted retroactively within 2 years of the month in which medical services were provided. In addition, presenting a “Certificate of Application of Maximum Amount” at the time of hospitalization limits the initial payment to the ceiling amount, thereby reducing the immediate financial burden.

Certain chronic conditions requiring long-term, high-cost treatments may qualify for special provisions that reduce the monthly ceiling to ¥10,000 (e.g., hemophilia, dialysis, and HIV infection) for all individuals.

## History and Current Discussion

In 1961, Japan implemented universal health coverage, under which all citizens were required to join the National Health Insurance Association established by municipalities, employee health insurance, or a mutual aid association, as mandated by law ^(6)^. Nevertheless, households faced serious financial strain because no maximum limit was set on out-of-pocket expenses.

In 1973, the Japanese government established the High-Cost Medical Expense Benefit System through legislative amendments. Household aggregation, multimonthly ceilings, and classification by age and income began in 1984. Under this new system, patients could receive reimbursement by applying to their insurers after paying their total out-of-pocket medical expenses (i.e., the reimbursement system). However, this payment often still imposed a temporary, excessive financial burden on patients. Consequently, since 2007, upon prior application to the insurer, patients have only been required to pay either their standard co-payment or the monthly ceiling amount, whichever is less (i.e., an in-kind benefit system) (Figure 1). In 2012, a similar system was introduced for outpatient medical expenses. In 2021, an online qualification verification system was developed that enabled patients to scan their Individual Number Cards to receive in-kind benefits without prior application ^(7)^.

A High-Cost Medical Expense Benefit System may prevent individuals from incurring catastrophic healthcare expenditures, often observed in middle- and low-income countries ^(8)^. A large-scale global analysis from 133 countries reported that the incidence of catastrophic health expenditure, defined as out-of-pocket spending exceeding 10% of household consumption, increased from 9.7% in 2000 to 11.7% in 2010 ^(8)^. In Japan, analyses using nationally representative household panel data between 2004 and 2020 showed that approximately 9.8% of households experienced catastrophic health expenditure at the same threshold ^(9)^. When facing catastrophic healthcare expenditures, Japanese patients aged ≥65 years were likely to reduce food expenditures, while those aged <65 years were likely to reduce education expenditures ^(9)^; these observations highlight the age-specific variables that can affect long-term health outcomes. Furthermore, although these studies did not identify the underlying diseases, the economic burden is particularly pronounced among patients with malignant diseases ^(10), (11)^. Therefore, the High-Cost Medical Expense Benefit System would be essential for patients, especially those with cancer, to receive optimal medical care while still maintaining their normal lifestyles.

From a health financing perspective, Japan’s High-Cost Medical Expense Benefit System can be understood as a mechanism that prioritizes financial protection through extensive risk pooling ^(12)^. Income-based monthly ceilings and household aggregation are consistent with the principle of vertical equity, as they limit out-of-pocket payments according to patients’ ability to pay and concentrate protection on those facing high medical costs (e.g., catastrophic healthcare expenditures). At the same time, this design entails inherent trade-offs with efficiency and fiscal sustainability. By substantially reducing marginal cost sharing once the ceiling is reached, the system may weaken price signals for high-cost care and shift a larger share of expenditure to the insurance pool. These trade-offs provide important context for understanding subsequent policy debates over the appropriate level of cost sharing in a universal health coverage system.

The high cost of new drugs and medical technologies has indeed strained Japan’s universal health insurance system, compounded by a declining birth rate and an aging population. Thus, in the winter of 2024, the Japanese government proposed an amendment to raise the monthly ceiling of the High-Cost Medical Expense Benefit System that would be phased in over 2 years. As the average salary growth rate was between 9.5% and 12.0% over the approximately 10 years before the amendment, the government proposed raising the upper limit by 10.0% for households with an average income. The amendment was estimated to reduce annual national medical expenditure by ¥370 billion ^(13)^. The government proposed an amendment to the limit presumably because the limit is specified only by cabinet order, and its amendment allows for faster policy adjustment; that is, an amendment to the co-payment rate (particularly those affecting the elderly, a large segment of the electorate) stipulated by the Health Insurance Law would require time-consuming Diet deliberation ^(14)^.

However, the proposal was strongly criticized because the opinions of patient organizations had not been considered. From a stakeholder perspective, the proposed increase in the monthly ceiling was expected to improve fiscal sustainability for insurers and the government, while increasing out-of-pocket costs for patients receiving long-term, high-cost treatments, such as those for cancer and intractable diseases. The government subsequently decided to withdraw the proposal and established a committee to discuss the future of the system by the winter of 2025. The committee, comprising relevant organizations and experts, began discussions based on the following perspectives: ensuring intergenerational equity, building sustainability, promoting the health of the working-age population, and delivering effective benefits ^(15)^. Consequently, the government decided to raise the monthly ceiling while maintaining a multimonthly ceiling and establishing an annual upper limit in 2026 ^(16)^. This amendment addresses the concerns of patients requiring long-term and high-cost treatments; however, the exclusion of no-value and low-value care from insurance coverage ^(17)^, the equalization of co-payment rates irrespective of age, and the introduction of asset-based co-payment may represent additional policy options to improve the sustainability of Japan’s universal health insurance system. The High-Cost Medical Expense Benefit System is expected to maintain opportunities for patients across all generations to receive advanced and high-cost medical care without incurring catastrophic medical expenditures.

## Conclusion

Japan’s High-Cost Medical Expense Benefit System exemplifies a structured approach to mitigate the financial impact of medical expenses on individuals. Reimbursement thresholds determined by age and income, along with household aggregation and multimonthly ceilings, can ensure equitable access to healthcare services.

## Article Information

### Author Contributions

Takaaki Konishi, Kimiko Hirata, Mio Nakata, and Tsuguo Iwatani designed and conceived this study. Takaaki Konishi collected the data and drafted the manuscript. Tsuguo Iwatani acquired funding for the study. All authors reviewed and edited the manuscript and approved the final manuscript.

### Conflicts of Interest

Takaaki Konishi received grants from Pfizer Health Research Foundation and research funds under contract from Boston Medical Sciences and had an advisory role in Health Insurance Claims Review and Reimbursement Services. Eriko Tokunaga received honoraria (lecture fee) from Eli Lilly, Daiichi Sankyo, Astra Zeneca, Chugai, and Gilead Sciences. The other authors have no conflicts of interest to declare.
